# Association of in-hospital resource utilization with post-acute spending in Medicare beneficiaries hospitalized for acute myocardial infarction: a cross-sectional study

**DOI:** 10.1186/s12913-019-4018-0

**Published:** 2019-03-25

**Authors:** Sudhakar V. Nuti, Shu-Xia Li, Xiao Xu, Lesli S. Ott, Tara Lagu, Nihar R. Desai, Karthik Murugiah, Michael Duan, John Martin, Nancy Kim, Harlan M. Krumholz

**Affiliations:** 1 Yale School of Medicine, 333 Cedar Street, New Haven, CT 06516 USA; 2 Center for Outcomes Research and Evaluation, Yale-New Haven Hospital, 1 Church Street, Suite 200, New Haven, CT 06510 USA; 3 Department of Obstetrics, Gynecology, and Reproductive Sciences, Yale School of Medicine, 310 Cedar Street, LSOG 205B, New Haven, CT 06510 USA; 4 Tufts University School of Medicine, 145 Harrison Ave, Boston, MA USA; 5 Department of Medicine, Baystate Medical Center, 759 Chestnut Street, Springfield, MA 01199 USA; 6 Section of Cardiovascular Medicine, Department of Internal Medicine, Yale School of Medicine, 789 Howard Avenue, FMP 330, New Haven, CT 06520 USA; 7 Premier, Inc, 13034 Ballantyne Corporate Place, Charlotte, NC 28277 USA; 8 Section of General Internal Medicine, Department of Internal Medicine, Yale School of Medicine, 333 Cedar Street, New Haven, CT 06520 USA; 9 Department of Health Policy and Management, Yale School of Public Health, 60 College Street, New Haven, CT USA; 10 Present address: Remedy Partners, 1120 Boston Post Road, Suite 3, Darien, CT 06820 USA

**Keywords:** Medicare, Costs, Bundled payments, Post-acute, Health policy

## Abstract

**Background:**

Efforts to decrease hospitalization costs could increase post-acute care costs. This effect could undermine initiatives to reduce overall episode costs and have implications for the design of health care under alternative payment models.

**Methods:**

Among Medicare fee-for-service beneficiaries aged ≥65 years hospitalized with acute myocardial infarction (AMI) between July 2010 and June 2013 in the Premier Healthcare Database, we studied the association of in-hospital and post-acute care resource utilization and outcomes by in-hospital cost tertiles.

**Results:**

Among patients with AMI at 326 hospitals, the median (range) of each hospital’s mean per-patient in-hospital risk-standardized cost (RSC) for the low, medium, and high cost tertiles were $16,257 ($13,097–$17,648), $18,544 ($17,663–$19,875), and $21,831 ($19,923–$31,296), respectively. There was no difference in the median (IQR) of risk-standardized post-acute payments across cost-tertiles: $5014 (4295-6051), $4980 (4349-5931) and $4922 (4056-5457) for the low (*n* = 90), medium (*n* = 98), and high (*n* = 86) in-hospital RSC tertiles (*p* = 0.21), respectively. In-hospital and 30-day mortality rates did not differ significantly across the in-hospital RSC tertiles; however, 30-day readmission rates were higher at hospitals with higher in-hospital RSCs: median = 17.5, 17.8, and 18.0% at low, medium, and high in-hospital RSC tertiles, respectively (*p* = 0.005 for test of trend across tertiles).

**Conclusions:**

In our study of patients hospitalized with AMI, greater resource utilization during the hospitalization was not associated with meaningful differences in costs or mortality during the post-acute period. These findings suggest that it may be possible for higher cost hospitals to improve efficiency in care without increasing post-acute care utilization or worsening outcomes.

**Electronic supplementary material:**

The online version of this article (10.1186/s12913-019-4018-0) contains supplementary material, which is available to authorized users.

## Background

Although United States (U.S.) inpatient costs are declining over time [1], post-hospitalization costs have more than doubled since 2001 [2] and account for a large portion of U.S. health care spending growth [3]. As a result, the post-acute care period has become a focus of programs designed to reduce waste and control costs [4, 5]. The move towards payment models that extend beyond hospitalizations to the entire episode of a patient’s illness [6] has only increased the interest in understanding the relationship between resource utilization during a hospitalization and utilization during the post-acute care period.

Although prior studies on episodes of care have shown that post-acute care is a substantial contributor to spending variation and therefore could be a target for cost savings [3, 7–9], there have been few studies on the relationship between in-hospital and post-acute resource utilization [10]. Such information would be valuable, as it is plausible that cost reductions in the hospitalization period could affect spending in the post-acute period. For example, hospitals that provide higher intensity care and spend more during the hospitalization might have a beneficial effect on patients after they are discharged, reducing the amount of post-acute care resources required. In contrast, hospitals that spend less during the hospitalization may discharge patients who require greater post-acute care utilization. Alternatively, it is also possible that there is no appreciable relationship between in-hospital and post-acute resource utilization. Knowledge about the relationship between early and late use of resources may help inform strategies to reduce resource utilization in the post-acute period while maintaining or improving health outcomes.

To examine these issues, we studied Medicare fee-for-service beneficiaries 65 years of age or older who were hospitalized for acute myocardial infarction (AMI), a common condition associated with significant mortality and costs. Medicare beneficiaries are the focus of government efforts to assess new payment models [6] and for whom post-discharge payment and 30-day outcomes measures are constructed [11]. We examined the association between in-hospital and post-discharge resource utilization, compared patient outcomes across hospitals stratified according to their in-hospital costs, and assessed patterns of in-hospital and post-acute resource utilization across hospitals to understand how hospitals use their resources.

## Methods

### Data sources

We used the Premier Healthcare Database (PHD) by Premier Inc., Charlotte, North Carolina, which contains highly specific information on in-hospital administrative and operational data from a large network of hospitals and ancillary providers nationwide, representing more than 330 million discharges. PHD contains a date-stamped log of all billed items and resource utilization during hospitalizations (e.g., medications and laboratory, diagnostic, and therapeutic services), diagnosis and procedure codes, and patient and hospital characteristics [12, 13]. Patient-level data were de-identified in accordance with the Health Insurance Portability and Accountability Act.

We also utilized the Centers for Medicare & Medicaid Services (CMS) publicly reported measures on hospital-specific, 30-day risk-standardized payment (RSP), 30-day risk-standardized mortality rate (RSMR), and 30-day risk-standardized readmission rate (RSRR) for 2010–2013 for AMI [11]. Of note, the 30-day RSP and RSMR measures start at patient admission. In addition, we obtained hospital-specific data on average per-patient payment for various post-acute care settings after discharge from the index hospitalization for AMI such as emergency department, readmission, outpatient physician visits, and skilled nursing facility. The publicly reported data were linked to PHD by hospital ID. Notably, some hospitals in our cohort were not linkable to the CMS public reporting data as they did not have a CMS hospital ID, shared a hospital ID with another hospital, or did not report a given measure. In brief, the RSP measure is determined by calculating payments during the 30-day AMI episode of care after removing payment adjustments for policy initiatives and geographic factors to better reflect variation in resource utilization related to clinical care. Payments were then risk-standardized based on patient clinical characteristics in the AMI hospitalization and the 12 months before hospitalization [14, 15].

The study was conducted under a collaborative contract with Premier, Inc. regarding protection of privacy of hospitals and other providers, which permits the linking of hospital data. We do not report any results that would reveal hospital identities. The Yale University Human Investigation Committee exempted this study protocol as defined by the Office of Human Research Protections.

### Study population

We included hospitals that were revascularization-capable, had intensive care units (ICUs)/cardiac care units (CCUs), and had 25 or more eligible AMI hospitalizations between July 2010 and June 2013. The revascularization-capable hospitals are defined as those that performed ≥5 percutaneous coronary intervention (PCI) or coronary artery bypass grafting (CABG) procedures for AMI from 2010 to 2013.

An eligible AMI hospitalization is defined as one in which the patient was 65 years or older at admission with a principal diagnosis of new AMI, i.e., International Classification of Diseases, Ninth Revision, Clinical Modification (ICD-9-CM) codes 410.xx (except for 410.×2 (AMI, subsequent episode-of-care)) and was not transferred from or to another acute care facility, did not involve a heart transplant or left ventricular assist device implantation, and had a length of stay greater than 1 day (unless death occurred). The age restriction was chosen to be consistent with CMS publicly reported mortality, readmission, and payment measures.

### Hospital characteristics

Hospital characteristics were available from PHD, including: number of beds, teaching hospital status, service of a rural or urban population, and the U.S census region where a hospital is located.

### Hospital costs and outcomes

We measured in-hospital cost for each hospitalization, the methods of which are detailed elsewhere [13]. Briefly, we used hospital cost accounting data available in the Premier database and removed geographic variation in input prices by applying average unit cost across all hospitals for each service item by year. All cost estimates were adjusted to 2013 U.S. dollars. To reduce the influence of outliers, we Winsorized cost data within each year at the 0.5th and 99.5th percentiles. Hospital-level risk-standardized in-hospital RSC (hereafter in-hospital RSC) was calculated by adjusting for patient case-mix using hierarchical generalized linear models [13]. Candidate risk factors include patient age, comorbidities [16], prior revascularization procedures (CABG and PCI), and hospital transfer-in and transfer-out rate for all patients with AMI during the study period. Stepwise selection was used to select final risk-adjustors.

To assess resource utilization after hospitalization for AMI, we also estimated a risk-standardized, post-acute payment measure (post-acute RSP) for each hospital. Because PHD is de-identified, we could not link a patient’s hospitalization with his/her post-discharge care information and hence could not perform direct risk adjustment for the post-acute payment measure. Therefore, we used a “pseudo” risk-standardization approach via prorating. Specifically, for each hospital, using CMS data, we first calculated its average per patient post-acute care payment (not risk-standardized) and the proportion of its total 30-day episode of care payment (not risk-standardized) that occurred after discharge from the index hospitalization. Then we multiplied the hospital’s CMS publicly reported 30-day RSP (risk-standardized) by this proportion, and used this estimate as our measure for post-acute RSP. For example, for a hospital, its 30-day RSP is $15,000, and its unadjusted average per patient in-hospital and post-acute payments are $18,000 and $6000, respectively. We then calculate the post-acute RSP for this hospital as 15,000 * [6000/(18,000 + 6000)] = $3750.

In addition, we assessed in-hospital mortality for each hospitalization. Hospital-level risk-standardized, in-hospital mortality (in-hospital RSMR) was estimated using hierarchical generalized linear models with adjustment for patient case-mix. We also linked hospitals in our sample to 2010–2013 CMS hospital-specific public reporting data on 30-day RSMRs and 30-day RSRRs, as PHD is de-identified and we could not measure post-discharge outcomes for individual hospitalizations.

### Hospital practice

For each hospitalization, we examined whether the patient was admitted to an ICU, the length of ICU/CCU stay in days, and the overall length of hospital stay; all hospitals in our sample had ICUs and CCUs. We also examined whether patients received any interventional cardiac therapies, such as cardiac catheterization, CABG surgery, PCI procedures, or intra-aortic balloon pump (IABP). Receipt of revascularization was determined based on ICD-9-CM procedure codes and hospital charge codes for PCI or CABG services (Additional file 1: Appendix A). Receipt of IABP was based on ICD-9-CM code 37.61. Use of ICU care, number of ICU days, and cardiac catheterizations were identified based on hospital charge codes (see Additional file 1: Appendix B for list of codes).

### Statistical analysis

Hospitals were divided into tertiles based on in-hospital RSC, namely low, medium, and high risk-standardized cost tertiles. We utilized frequency (proportion) to characterize categorical variables, and median (interquartile range: IQR) or mean (standard deviation: SD) to characterize continuous variables. Differences in patient and hospital characteristics across in-hospital RSC tertiles were assessed using chi-square tests for categorical variables and Kruskal-Wallis tests for continuous variables. In addition to assessing the post-acute RSP by in-hospital RSC tertiles, as a sensitivity analysis we assessed the Pearson correlation between the in-hospital RSC and post-acute RSP as another approach.

We also calculated and compared mean per-patient cost in various in-hospital cost departments (e.g., cardiac procedures, ICU/CCU, laboratory) and per-patient payment in different post-acute care settings (e.g., emergency department, skilled nursing facility (SNF), readmission) by in-hospital risk-standardized cost tertiles. We used Wilcoxon analyses to test whether there is overall association between mean cost in each cost department or post-acute care setting and in-hospital RSC tertiles. We used the Dwass, Steel, Critchlow-Fligner (DSCF) method for multiple comparison analysis [17–19]. We also assessed the association between in-hospital ICU/CCU use and post-discharge SNF payment by regressing logarithmic hospital average SNF payment over hospital ICU/CCU use rate (proportion of patients using ICU/CCU) and average days in ICU/CCU, because they are settings with higher, variable resource utilization [20]. We conducted a sensitivity analysis to determine whether vital status contributed to differences in post-acute payments. *P*-values < 0.05 were deemed statistically significant. Analyses were performed using SAS version 9.4.

Our study has limitations. First, it is a cross-sectional study, so we cannot draw causal inferences regarding whether change in in-hospital RSCs would affect post-hospital payments. Future studies with longitudinal data would help provide additional insights. Second, our findings may not be broadly generalizable because we studied Medicare fee-for-service beneficiaries ≥65 years of age at 326 hospitals within the PHD. Third, there are limitations in using claims data for measurement of risk factors and risk standardization, but we utilized established risk models for adjustment that have been validated for claims [14, 21, 22]. Fourth, due to the de-identified nature of our data, we could not track resource utilization or outcomes for individual patients after discharge from the initial hospitalization. Therefore, we relied on CMS publicly reported measures to approximate longer-term patient outcomes and post-discharge resource utilization. We also had to use hospital-level post-acute payment data instead of patient-level linked in-hospital and post-acute cost data. Although this approach may not accurately reflect patient outcomes in our analytic sample or the exact costs of post-acute care, it should provide a reasonable approximation to help understand patterns of care and resource utilization. Furthermore, we were not able to link all of the Premier hospitals to CMS measures, which may affect the external validity of the study. Finally, we measured resource utilization via hospital costs for inpatient care and payments for post-acute care. While we recognize that they represent 2 different perspectives (provider vs. payer) of the burden on our healthcare system, they are a fair representation of the resources consumed in the respective settings.

## Results

### Variation in Total in-hospital RSC

Initially, there were 123,002 AMI patients in 326 hospitals in our cohort; after excluding non-Medicare patients and hospitals with less than 25 AMI patients during the study period, 82,008 patients hospitalized with AMI at 326 eligible hospitals remained (Additional file 1: Figure S1). There were 108 hospitals (24,799 patients with AMI) in the low in-hospital RSC tertile, 109 hospitals (28,198 patients) in the medium in-hospital RSC tertile, and 109 hospitals (29,011 patients) in the high in-hospital RSC tertile (Table 1, Additional file 1: Table S1). The median (range) of each hospital’s mean per-patient in-hospital RSC for the low-, medium-, and high-cost tertiles was $16,257 ($13,097–$17,648), $18,544 ($17,663–$19,875), and $21,831 ($19,923–$31,296), respectively.

**Table 1:** Characteristics of hospitals in different tertiles of risk-standardized in-hospital cost

Hospital Characteristics	Overall	Risk-Standardized In-Hospital Cost Tertile			*p*-value
		Low ($13,097-17,648)	Medium ($17,663-19,875)	High ($19,923-31,296)	
Total no. of hospitals	326	108	109	109	
AMI 3-year volume					0.06
< 146 (low)	33.4	42.6	25.7	32.1	
146–284 (medium)	33.1	30.6	39.5	29.4	
> 284 (high)	33.4	26.9	34.9	38.5	
Beds					0.09
< 257 (low)	33.1	43.5	26.6	29.4	
257–409 (medium)	33.4	27.8	37.6	34.9	
> 409 (high)	33.4	28.7	35.8	35.8	
Teaching hospitals					0.60
Yes	34.7	35.2	31.2	37.6	
No	65.3	64.8	68.8	62.4	
Census regions					0.38
Midwest	24.9	31.5	22.9	20.2	
Northeast	13.5	12.0	11.9	16.5	
South	42.3	36.1	44.0	46.8	
West	19.3	20.4	21.1	16.5	
Population served					0.37
Urban	85.3	82.4	89.0	84.4	
Rural	14.7	17.6	11.0	15.6	

*AMI* Acute myocardial infarction

### Variation in components of in-hospital resource utilization

In terms of utilization, patients in higher in-hospital RSC hospitals appeared to receive more intensive care during the inpatient stay (Table 2). The proportion of patients receiving ICU care was 44.0, 46.7, and 58.6%, respectively, at low, medium, and high in-hospital RSC hospitals (*p* < 0.001); and the IQRs for number of days in ICUs were 1–3, 1–4, and 2–5 (*p* < 0.001), respectively. Similarly, patients in the higher in-hospital RSC hospitals were more likely to receive interventional therapies, such as proportion of patients receiving cardiac catheterization, CABG, PCI, and IABP (*p* < 0.001 for all). Median length of stay in days (IQR) was 4 (2–6), 4 (3–7), and 4 (3–8) across in-hospital RSC tertiles (*p* < 0.001).

**Table 2:** Utilization characteristics of patients hospitalized with acute myocardial infarction by hospital risk-standardized in-hospital cost levels

Utilization Characteristics	Overall	Risk-Standardized In-Hospital Cost Tertile			*p*-value
		Low ($13,097-17,648)	Medium ($17,663-19,875)	High ($19,923-31,296)	
Total no. of hospitals	326	108	109	109	
Selected inpatient care during index episode					
Index episode length of stay, median (IQR), days	4 (3–7)	4 (2–6)	4 (3–7)	4 (3–8)	< 0.001
ICU admission during index episode, %	50.1	44.0	46.7	58.6	< 0.001
ICU days if admitted, median (IQR)	2 (1–4)	2 (1–3)	2 (1–4)	3 (2–5)	< 0.001
Interventional cardiac therapies (%)					
Cardiac catheterization	63.9	61.4	64.9	64.9	< 0.001
CABG surgery	7.8	6.9	8.0	8.5	< 0.001
Percutaneous coronary intervention	41.4	40.1	41.2	42.8	< 0.001
Intra-aortic balloon pump	4.7	4.0	4.7	5.3	< 0.001

*CABG* Coronary artery bypass graft, *ICU* Intensive care unit, *IQR* Interquartile range

**Fig. 1:**
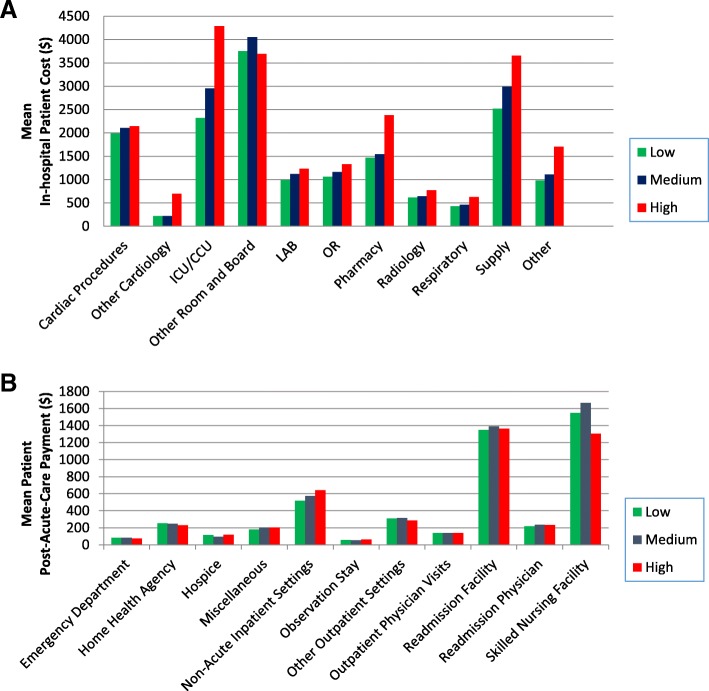
**a** and **b**. Resource Utilization by In-Hospital Cost Tertiles. **a** Relative Contribution of Service Categories to In-Hospital Costs by Hospitals in Different In-Hospital Cost Tertiles. Green bars indicate the low in-hospital cost tertile, blue bars the medium in-hospital cost tertile, and red bars the high in-hospital cost tertile. ICU: intensive care unit; CCU: coronary care unit; OR: operating room. **b** Relative Contribution of Service Categories to Post-Acute Payments by Hospitals in Different In-Hospital Cost Tertiles. Green bars indicate the low in-hospital cost tertile, blue bars the medium in-hospital cost tertile, and red bars the high in-hospital cost tertile

The departments with the greatest resource utilization measured by per person mean spending were ICU/CCU, other room and board, supply, and cardiac procedures for all 3 in-hospital RSC tertiles (Fig. 1a, Additional file 1: Tables S2 and S3, Additional file 1: Figures S2‑S4). However, departments that varied the most across low, medium, and high in-hospital RSC tertiles were ICU/CCU, supplies, and pharmacy. The high in-hospital RSC hospitals spent notably more on ICU/CCU and pharmacy than other hospitals: $2339, $2876 and $4285 per person for ICU/CCU spending and $1408, $1572, and $2578 per person for pharmacy spending, for the low, medium, and high in-hospital RSC hospital tertiles, respectively. Mean per-patient spending on cardiac procedures, however, was similar among the 3 tertiles ($2008, $2153, and $2074, respectively).

### Variation in hospital characteristics and outcomes

There were no significant differences in hospital characteristics across the 3 in-hospital RSC tertiles (Tables 1 and 2). There was also no significant difference in in-hospital RSMRs (Table 3). Of the 326 eligible hospitals in our cohort, 276 and 277 were successfully linked to the CMS 30-day RSMR and RSRR measures, respectively. There was no statistically significant difference in 30-day RSMRs among the in-hospital RSC tertiles, but 30-day RSRRs differed significantly across the in-hospital RSC tertiles (*p* = 0.03). Hospitals in tertiles with higher in-hospital RSC had higher RSRRs: the medians (IQRs) of RSRRs were 17.5% (16.8–18.4%), 17.8% (17.1–18.6%), and 18.0% (17.5–18.6%), respectively, for low, medium, and high in-hospital RSC tertiles, and the trend test treating in-hospital RSC tertile ranks as numeric variables had a *p*-value of 0.005.

**Table 3:** Outcomes and post-acute care costs of patients hospitalized with acute myocardial infarction by hospital risk-standardized in-hospital cost levels

Variable	Total	Inpatient Cost Level			*p*-value
		Low ($13,097-17,648)	Medium ($17,663-19,875)	High ($19,923-31,296)	
Outcomes					
In-hospital mortality rate	8.2 (7.9–8.8)	8.2 (7.8–8.7)	8.3 (7.8–8.8)	8.2 (7.8–8.7)	0.90
30-day risk-standardized mortality rate (*N* = 276)^a^, median (IQR)	14.7 (13.7–15.7)	14.9 (13.9–15.7)	14.6 (13.7–15.6)	14.7 (13.6–15.5)	0.42
30-day risk-standardized readmission rate (*N* = 277)^a^, median (IQR)	17.8 (17.1–18.5)	17.5 (16.8–18.4)	17.8 (17.1–18.6)	18.0 (17.5–18.6)	0.03
Hospital risk-standardized post-acute care payment (*N* = 274) ($), median (IQR)	4964 (4250–5931)	5014 (4295–6051)	4980 (4349–5931)	4922 (4056–5457)	0.21

^a^Data provided for the number of hospitals (N) that could be linked to Centers for Medicare & Medicaid Services measures. By cost level, for risk-standardized mortality rates, there were 89, 99, and 88 hospitals; for risk-standardized readmission rates, 90, 99, and 88 hospitals; for risk-standardized post-acute care payment, 90, 98, and 86 hospitals

Statistics are percent unless stated otherwise

### Relationship between in-hospital and post-acute care resource utilization

Of the 274 hospitals that were linked to the CMS AMI payment measure, there was no statistically significant difference in risk-standardized post-acute payments across in-hospital RSC tertiles: median (IQR) = $5014 (4295–6051), $4980 (4349–5931) and $4922 (4056–5457) for the low (*n* = 90), medium (*n* = 98), and high (*n* = 86) in-hospital RSC tertiles, respectively (*p* = 0.21) (Table 3). However, there was a modest yet statistically significant negative correlation between in-hospital RSC and risk-standardized post-acute care payments (correlation coefficient = − 0.13, *p* = 0.04, Additional file 1: Figure S5). On average, every $1000 increase in in-hospital RSC was associated with $50 less post-acute care payments (*p* = 0.04).

Three settings displayed the highest mean per-patient post-acute care payments: readmission, SNFs, and non-acute inpatient care (Additional file 1: Table S5, Fig. 1b); these findings were consistent in a sensitivity analysis stratified by vital status (Additional file 1: Table S4, Additional file 1: Figures S6a and b).

For the low, medium and high in-hospital RSC tertiles, respectively, the mean per-patient post-acute payments were $1534, $1633, and $1596 for readmissions (*p* = 0.001), $1535, $1542, and $1303 for SNFs (*p* < 0.001), and $533, $649, and $714 for the non-acute inpatient setting (*p* < 0.001). The highest in-hospital RSC tertile had lower SNF and home health agency use and payments, with spending in other post-acute settings similar across cost tertiles (Additional file 1: Tables S5 and S6, Additional file 1: Figures S7‑S9).

### Relationship between in-hospital ICU use and post-acute care resource utilization on SNF

Given the variation in both ICU utilization and SNF post-acute payments across in-hospital RSC tertiles, we assessed the association between ICU use rate and the logarithm of mean payments for SNF utilization among the 274 hospitals. For every 10% increase in use of ICUs, there was a 0.7% decrease in SNF payment (*p* < 0.003). Moreover, for each additional day in ICU, there was a 13% decrease in SNF payment (*p* < 0.002).

## Discussion

In our study of patients hospitalized with AMI, greater resource utilization during the hospitalization was not associated with substantially lower resource utilization during the post-acute period. Hospitals with higher in-hospital RSC spent more on ICU/CCU stays, pharmacy, and supplies, and had lower payments for SNFs and home health agencies. Across in-hospital RSC tertiles, hospitals had similar in-hospital and 30-day mortality rates, but those in the highest in-hospital RSC tertile had higher 30-day readmission rates. Although patients at hospitals with greater inpatient resource utilization were more likely to receive cardiac procedures, there were no marked differences in other treatment characteristics across in-hospital RSC tertiles.

Prior research on bundled and episode-of-care payments shows that post-acute care is a substantial contributor to spending variation [3, 7–9]. Our work extends this literature as the first large, contemporary study to assess the association of resource utilization patterns between in-hospital and post-acute care. A previous study analyzing patient-level data showed that among Medicare beneficiaries with AMI, patient disease severity at hospital discharge, discharging hospital for-profit ownership, and discharging hospital provision of home health services were important predictors of post-acute service utilization; however, the study did not have access to detailed in-hospital cost data and the ability to study in-hospital RSCs or post-acute spending, or conduct a hospital-level analysis [23]. Another study of patients with heart failure and pneumonia showed that those initially seen in lower cost hospitals had lower spending associated with readmission within 6 months of the index hospitalization, but the study did not assess spending across the various post-acute care settings [24].

Several studies have assessed the association between hospital spending and outcomes, but the results have been mixed [25–31]. Our study adds to the literature that shows no association between increased hospital resource utilization and better outcomes. In fact, readmission was highest in hospitals with higher in-hospital resource utilization. Further research is needed to determine whether spending reduction is a means to improve outcomes.

There are several possible explanations for our findings. There may be a core set of services that are necessary to ensure that people have better outcomes and lower post-acute utilization that are provided by all hospitals, and the increased resource utilization by high-cost hospitals may be in lower yield areas. Second, there may be certain resource utilization “habits” in higher spending hospitals that inflate costs and could be curtailed. For example, one study in the Premier Healthcare Database has shown that some hospitals may perform more angiographies that ultimately do not result in higher rates of revascularization, which may partly explain the greater utilization in those hospitals [32]. Moreover, studies have shown that PCI procedures are associated with increased readmission rates [33], with many of these readmissions being preventable [34], which may contribute to the higher readmission rates in our study. Nonetheless, the prior cited study using the PHD found no differences in readmissions [32], with hospitals that do more being unlikely to have a lower threshold of readmission, and there is a large range of use that is not associated with outcomes [20, 35]. We could not, with this design, explain why higher costs may be associated with higher rates of readmission. If the relationship is true, and not confounded, it may be related to the fact that excess tests, procedures, and medications increase the likelihood of adverse events. In addition, longer length of stay and intensity of the stay, including use of the intensive care unit, could provide more exposure to the allostatic stress of the hospitalization, leading to greater downstream vulnerability [36]. It may be that there is a sweet spot of intensity for each patient, and places that tend to do more may compromise downstream recovery. It is also possible that higher cost hospitals have stricter definitions of AMI and therefore have higher in-hospital utilization rates and, given their severity, patients may be more likely to be readmitted, though the magnitude of differences at the hospital level suggests that differences in severity alone are unlikely to explain our findings. Finally, there may be other determinants of post-acute care utilization that were not captured in our study, an important area for future research. For example, through linking patients in the in-hospital and post-acute settings, one might explore which particular categories of in-hospital expenditures are associated with the risk for greater utilization of post-discharge services – and whether they are markers for higher risk of utilization or something that mediates the needs for greater utilization. In particular, there is a need for studies surrounding how the length and intensity of the hospitalization relates to the recovery after discharge.

Reducing unnecessary post-acute resource utilization is a goal as the government and health care industry try to contain costs and improve the value of care, and new models of payment that focus on the entire episode of patient care, such as the Bundled Payments for Care Improvement Initiative [6], are an important first step. Our study suggests the absence of a strong association between higher in-hospital resource utilization and reductions in post-discharge resource utilization. Furthermore, post-hospitalization outcomes were not better. These findings suggest that greater resource utilization at the higher cost hospitals may fail to provide a meaningful benefit for patients and the health system. Moving forward, higher spending hospitals may target areas of higher cost during a hospitalization without increasing post-acute care utilization or worsening outcomes. The insight, however, requires the ability to benchmark.

## Conclusions

In our study of a large number of hospitals across the country, greater resource utilization for patients hospitalized with AMI during the inpatient stay was not associated with substantially lower 30-day post-acute care resource utilization or better outcomes. In the context of novel care and payment models that extend across the entire episode of care, where entities will be rewarded for reducing spending, our findings suggest that it may be possible for higher cost hospitals to improve efficiency in care without increasing post-acute care utilization or jeopardizing outcomes of care.

## Additional file

Additional file 1: **Appendix A.** List of ICD-9 and Standard Charge Codes for Identifying Percutaneous Coronary Intervention and Coronary Artery Bypass Graft Procedures. Appendix B. Standard Charge Codes for Identifying ICU/CCU and Catheterization. **Table S1.** Characteristics of Patients Hospitalized with Acute Myocardial Infarction by Hospital Risk-Standardized In-Hospital Cost Levels. **Table S2.** In-Hospital Resource Utilization: Mean Per-Patient Cost in Each Department. **Table S3.** In-Hospital Resource Utilization: Mean per Patient Number of Items. **Table S4.** Patient Costs by In-Hospital Cost Ranks and Vital Status. **Table S5.** Mean Per Patient Post-Acute-Care Payment at Different Care Settings. **Table S6.** Percentage of Patients Using Various Post-Acute-Care Services by Cost Groups. **Figure S1.** Flowchart of Exclusion Criteria. **Figure S2.** Number of Items Ordered during a Hospitalization by In-Hospital Cost Tertile. **Figure S3.** Mean In-hospital Resource Utilization per User by In-Hospital Cost Tertiles. **Figure S4.** Relative Contribution of Service Categories to In-Hospital Costs by In-Hospital Cost Tertiles. **Figure S5.** Correlation Between Hospital Risk-Standardized In-Hospital Cost ($) and Hospital Risk-Standardized Post-Acute Payments ($). **Figure S6.a** In-Hospital Spending Among Survivors. **b** In-Hospital Spending Among the Expired. **Figure S7.** Percentage of Patients Using Different Post-Acute-Care Services by In-Hospital Cost Tertiles. **Figure S8.** Mean User Post-Acute Payments by In-hospital Cost Tertiles. **Figure S9.** Relative Contribution of Service Categories to Post-Acute Payments by In-Hospital Cost Tertiles. (DOCX 178 kb)
