# Supplementary material for: Association of in-hospital resource utilization with post-acute spending in Medicare beneficiaries hospitalized for acute myocardial infarction: a cross-sectional study
Source: BMC Health Serv Res. 2019 Mar 25;19:190. doi: 10.1186/s12913-019-4018-0 (PMC6432744; doi:10.1186/s12913-019-4018-0)
Supplement: Supplementary file 1 — Appendix A. List of ICD-9 and Standard Charge Codes for Identifying Percutaneous Coronary Intervention and Coronary Artery Bypass Graft Procedures. Appendix B. Standard Charge Codes for Identifying ICU/CCU and Catheterization. Table S1. Characteristics of Patients Hospitalized with Acute Myocardial Infarction by Hospital Risk-Standardized In-Hospital Cost Levels. Table S2. In-Hospital Resource Utilization: Mean Per-Patient Cost in Each Department. Table S3. In-Hospital Resource Utilization: Mean per Patient Number of Items. Table S4. Patient Costs by In-Hospital Cost Ranks and Vital Status. Table S5. Mean Per Patient Post-Acute-Care Payment at Different Care Settings. Table S6. Percentage of Patients Using Various Post-Acute-Care Services by Cost Groups. Figure S1. Flowchart of Exclusion Criteria. Figure S2. Number of Items Ordered during a Hospitalization by In-Hospital Cost Tertile. Figure S3. Mean In-hospital Resource Utilization per User by In-Hospital Cost Tertiles. Figure S4. Relative Contribution of Service Categories to In-Hospital Costs by In-Hospital Cost Tertiles. Figure S5. Correlation Between Hospital Risk-Standardized In-Hospital Cost ($) and Hospital Risk-Standardized Post-Acute Payments ($). Figure S6.a In-Hospital Spending Among Survivors. b In-Hospital Spending Among the Expired. Figure S7. Percentage of Patients Using Different Post-Acute-Care Services by In-Hospital Cost Tertiles. Figure S8. Mean User Post-Acute Payments by In-hospital Cost Tertiles. Figure S9. Relative Contribution of Service Categories to Post-Acute Payments by In-Hospital Cost Tertiles. (DOCX 178 kb) [file 12913_2019_4018_MOESM1_ESM.docx]

Additional file 1

**Association of In-Hospital Resource Utilization with Post-Acute Spending in Medicare Beneficiaries Hospitalized with Acute Myocardial Infarction**

**Table of Contents**

eAppendix A. List of ICD-9 and Standard Charge Codes for Identifying Percutaneous Coronary Intervention and Coronary Artery Bypass Graft Procedures 2

eAppendix B. Standard Charge Codes for Identifying ICU/CCU and Catheterization 8

eTable 1. Characteristics of Patients Hospitalized with Acute Myocardial Infarction by Hospital Risk-Standardized In-Hospital Cost Levels 12

eTable 2. In-Hospital Resource Utilization: Mean Per-Patient Cost in Each Department 14

eTable 3. In-Hospital Resource Utilization: Mean per Patient Number of Items Ordered in Each Department 15

eTable 4. Patient Costs by In-Hospital Cost Ranks and Vital Status 16

eTable 5. Mean Per Patient Post-Acute-Care Payment at Different Care Settings 17

eTable 6. Percentage of Patients Using Various Post-Acute-Care Services by Cost Groups 18

eFigure 1. Flowchart of Exclusion Criteria 19

eFigure 2. Number of Items Ordered during a Hospitalization by In-Hospital Cost Tertiles 20

eFigure 3. Mean In-hospital Resource Utilization per User by In-Hospital Cost Tertiles 21

eFigure 4. Relative Contribution of Service Categories to In-Hospital Costs by In-Hospital Cost Tertiles 22

eFigure 5. Correlation Between Hospital Risk-Standardized In-Hospital Cost ($) and Hospital Risk-Standardized Post-Acute Cost ($) 23

eFigure 6a. In-Hospital Spending Among Survivors 24

eFigure 6b. In-Hospital Spending Among the Expired 25

eFigure 7. Percentage of Patients Using Different Post-Acute-Care Services by In-Hospital Cost Tertiles 26

eFigure 8. Mean User Post-Acute Payments by In-hospital Cost Tertiles 27

eFigure 9. Relative Contribution of Service Categories to Post-Acute Payments by In-Hospital Cost Tertiles 28

# Appendix A. List of ICD-9 and Standard Charge Codes for Identifying Percutaneous Coronary Intervention and Coronary Artery Bypass Graft Procedures.

ICD-9 and standard charge codes for identifying PCI

ICD-9 codes: 00.66, 36.01, 36.02, and 36.05-36.07

PCI standard charge code and descriptions

| **Standard Charge Code** | **Standard Charge Descriptions** |
| --- | --- |
| 270270008910000 | CATHETER ATHERECTOMY (CUTTING BALLOON) |
| 270270008970000 | CATHETER BALLOON ACE |
| 270270008980000 | CATHETER BALLOON ACE LONG |
| 270270008990000 | CATHETER BALLOON ANGIOGRAM |
| 270270009000000 | CATHETER BALLOON ARC |
| 270270009010000 | CATHETER BALLOON ASSIST |
| 270270009020000 | CATHETER BALLOON BAILOUT CATH LAB |
| 270270009030000 | CATHETER BALLOON BANDIT |
| 270270009050000 | CATHETER BALLOON COBRA |
| 270270009070000 | CATHETER BALLOON DILATION CARDIAC |
| 270270009120000 | CATHETER BALLOON ELIPSE |
| 270270009150000 | CATHETER BALLOON EVERGREEN |
| 270270009180000 | CATHETER BALLOON GRAFT ACE |
| 270270009190000 | CATHETER BALLOON GRUNTZIG |
| 270270009220000 | CATHETER BALLOON MEDITECH |
| 270270009230000 | CATHETER BALLOON MILLENIA |
| 270270009240000 | CATHETER BALLOON MONGOOSE |
| 270270009260000 | CATHETER BALLOON NC RANGER |
| 270270009300000 | CATHETER BALLOON PERC |
| 270270009310000 | CATHETER BALLOON PMC |
| 270270009320000 | CATHETER BALLOON PREDATOR |
| 270270009330000 | CATHETER BALLOON PRISM |
| 270270009340000 | CATHETER BALLOON PROBE |
| 270270009350000 | CATHETER BALLOON PTCA |
| 270270009360000 | CATHETER BALLOON RALLY |
| 270270009370000 | CATHETER BALLOON RANGER |
| 270270009390000 | CATHETER BALLOON RX COMET |
| 270270009400000 | CATHETER BALLOON RX COMET VP |
| 270270009410000 | CATHETER BALLOON SAVVY |
| 270270009420000 | CATHETER BALLOON SLALOM |
| 270270009430000 | CATHETER BALLOON SURPASS |
| 270270009440000 | CATHETER BALLOON THOR |
| 270270009450000 | CATHETER BALLOON TITAN |
| 270270009460000 | CATHETER BALLOON TITAN MEGA |
| 270270009470000 | CATHETER BALLOON TRAKSTAR |
| 270270009480000 | CATHETER BALLOON TRAPPER |
| 270270009490000 | CATHETER BALLOON TX 2000 |
| 270270009500000 | CATHETER BALLOON ULTRA THICK |
| 270270009520000 | CATHETER BALLOON VALOR |
| 270270009530000 | CATHETER BALLOON VALVULOPLASTY |
| 270270010980000 | CATHETER GUIDING PTCA |
| 270270015340000 | CONNECTOR PTCA |
| 270270026270000 | GUIDEWIRE PTCA |
| 270270026280000 | GUIDEWIRE PTCA STEERABLE |
| 270270028380000 | INTRODUCER PTCA |
| 270270028400000 | INTRODUCER STENT SET |
| 270270031150000 | KIT PTCA |
| 270270031160000 | KIT PTCA GUIDEWIRE |
| 270270035010000 | PACK CATHETERIZATION LAB |
| 270270045670000 | STENT CORONARY |
| 270270045750000 | STENT INTRACORONARY |
| 270270095120000 | CATHETER BALLOON DISPATCH |
| 270270095140000 | CATHETER BALLOON LIFESTREAM |
| 270270095150000 | CATHETER BALLOON MAGNUM |
| 270270101030000 | CATHETER BALLOON UT 8MMX3 |
| 270270101040000 | CATHETER BALLOON BANDIT 3.75 NC |
| 270270101050000 | CATHETER BALLOON 2.5 |
| 270270101060000 | CATHETER BALLOON 2.5M |
| 270270101070000 | CATHETER BALLOON 3.0M |
| 270270101080000 | CATHETER BALLOON 3.5MM |
| 270270101090000 | CATHETER BALLOON 4MX2CM |
| 270270101100000 | CATHETER BALLOON 5FR 4X10 |
| 270270101110000 | CATHETER BALLOON 5FR |
| 270270101120000 | CATHETER BALLOON 6MM |
| 270270101130000 | CATHETER BALLOON 6X2 |
| 270270101140000 | CATHETER BALLOON 6X4 |
| 270270101150000 | CATHETER BALLOON 7X4 |
| 270270101160000 | CATHETER BALLOON 8FR |
| 270270101170000 | CATHETER BALLOON BMX 10X4 |
| 270270110060000 | STENT DRUG ELUTING |
| 270270110170000 | STENT CORDIS CYPHER DRUG ELUTING |
| 270270110190000 | STENT TAXUS EXPRESS PACLITAXEL-ELUTING |
| 270270111670000 | STENT MEDTRONIC ENDEAVOR |
| 270270112010000 | STENT CORONARY WITH DELIVERY SYSTEM |
| 480480000320000 | PTCA W/STENT EACH ADDL VESSEL |
| 480480000330000 | PTCA W/STENT SINGLE VESSEL |
| 480480000340000 | PTCA W/SUPPLIES |
| 480480929730000 | PERCUTANEOUS TRANSLUMINAL CORONARY THROMBECTOMY |
| 480480929800000 | STENT PLACEMENT SINGLE VESSEL |
| 480480929810000 | STENT PLACEMENT EACH ADDL VESSEL |
| 480480929820000 | PTCA SINGLE VESSEL |
| 480480929840000 | PTCA EACH ADDL VESSEL |

ICD-9 and standard charge codes for identifying CABG

CABG ICD-9 Code: 36.1, 36.10-36.17 and 36.19

CABG standard charge code and descriptions

| **Standard Charge Code** | **Standard Charge Descriptions** |
| --- | --- |
| 270270009620000 | CATHETER CABG |
| 270270034980000 | PACK CABG |
| 360360335080000 | ENDOSCOPY VASCULAR FOR VEIN HARVEST FOR CABG |
| 360360335100000 | CABG VEIN 1 GRAFT |
| 360360335110000 | CABG VEIN 2 GRAFTS |
| 360360335120000 | CABG VEIN 3 GRAFTS |
| 360360335130000 | CABG VEIN 4 GRAFTS |
| 360360335140000 | CABG VEIN 5 GRAFTS |
| 360360335160000 | CABG VEIN > 6 GRAFTS |
| 360360335170000 | CABG ARTERY/VEIN 1 GRAFT |
| 360360335180000 | CABG ARTERY/VEIN 2 GRAFTS |
| 360360335190000 | CABG ARTERY/VEIN 3 GRAFTS |
| 360360335210000 | CABG ARTERY/VEIN 4 GRAFTS |
| 360360335220000 | CABG ARTERY/VEIN 5 GRAFTS |
| 360360335230000 | CABG ARTERY/VEIN > 6 GRAFTS |
| 360360335300000 | REOPERATION CORONARY ARTERY BYPASS |
| 360360335330000 | CABG ARTERIAL 1 GRAFT |
| 360360335340000 | CABG ARTERIAL 2 GRAFTS |
| 360360335350000 | CABG ARTERIAL 3 GRAFTS |
| 360360335360000 | CABG ARTERIAL > 4 GRAFTS |
| 360360335420000 | MYCOCARDIAL RESECTION |
| 360360335720000 | ENDARTERECTOMY CORONARY OPEN |
| 360450335100000 | ER CABG VEIN 1 GRAFT |
| 360450335110000 | ER CABG VEIN 2 GRAFTS |
| 360450335120000 | ER CABG VEIN 3 GRAFTS |
| 360450335130000 | ER CABG VEIN 4 GRAFTS |
| 360450335140000 | ER CABG VEIN 5 GRAFTS |
| 360450335160000 | ER CABG VEIN > 6 GRAFTS |
| 360450335170000 | ER CABG ARTERY/VEIN 1 GRAFT |
| 360450335180000 | ER CABG ARTERY/VEIN 2 GRAFTS |
| 360450335190000 | ER CABG ARTERY/VEIN 3 GRAFTS |
| 360450335210000 | ER CABG ARTERY/VEIN 4 GRAFTS |
| 360450335220000 | ER CABG ARTERY/VEIN 5 GRAFTS |
| 360450335230000 | ER CABG ARTERY/VEIN > 6 GRAFTS |
| 360450335300000 | ER REOPERATION CORONARY ARTERY BYPASS |
| 360450335330000 | ER CABG ARTERIAL 1 GRAFT |
| 360450335340000 | ER CABG ARTERIAL 2 GRAFTS |
| 360450335350000 | ER CABG ARTERIAL 3 GRAFTS |
| 360450335360000 | ER CABG ARTERIAL > 4 GRAFTS |
| 970975335100000 | PF CABG VEIN 1 GRAFT |
| 970975335110000 | PF CABG VEIN 2 GRAFTS |
| 970975335120000 | PF CABG VEIN 3 GRAFTS |
| 970975335130000 | PF CABG VEIN 4 GRAFTS |
| 970975335140000 | PF CABG VEIN 5 GRAFTS |
| 970975335160000 | PF CABG VEIN > 6 GRAFTS |
| 970975335170000 | PF CABG ARTERY/VEIN 1 GRAFT |
| 970975335180000 | PF CABG ARTERY/VEIN 2 GRAFTS |
| 970975335190000 | PF CABG ARTERY/VEIN 3 GRAFTS |
| 970975335210000 | PF CABG ARTERY/VEIN 4 GRAFTS |
| 970975335220000 | PF CABG ARTERY/VEIN 5 GRAFTS |
| 970975335230000 | PF CABG ARTERY/VEIN > 6 GRAFTS |
| 970975335300000 | PF REOPERATION CORONARY ARTERY BYPASS |
| 970975335330000 | PF CABG ARTERIAL 1 GRAFT |
| 970975335340000 | PF CABG ARTERIAL 2 GRAFTS |
| 970975335350000 | PF CABG ARTERIAL 3 GRAFTS |
| 970975335360000 | PF CABG ARTERIAL > 4 GRAFTS |
| 999999041102008 | IMA GRAFT PERFORMED PRIMARY ISOLATED CABG |

# Appendix B. Standard Charge Codes for Identifying ICU/CCU and Catheterization.

ICU/CCU standard charge codes and descriptions

| **Standard Charge Code** | **Standard Charge Descriptions** |
| --- | --- |
| 110200000090000 | R&B ICU |
| 110200000420000 | R&B ICU ISOLATION |
| 110201000080000 | R&B SICU ISOLATION |
| 110201000260000 | R&B SICU |
| 110202000070000 | R&B MICU ISOLATION |
| 110202000120000 | R&B MICU |
| 110210000010000 | R&B CVICU |
| 110210000020000 | R&B CVICU ISOLATION |
| 110210000030000 | R&B CICU/CCU (CORONARY CARE) |
| 110210000060000 | R&B CICU/CCU ISOLATION (CORONARY CARE) |

Catheterization standard charge codes and descriptions

| **Standard Charge Code** | **Standard Charge Descriptions** |
| --- | --- |
| 270270008850000 | CATHETER ANGIOGRAM |
| 270270008860000 | CATHETER ANGIOGRAM 7FR 110CM |
| 270270008870000 | CATHETER ANGIOGRAM 7FR 90CM |
| 270270008880000 | CATHETER ANGIOGRAM 8FR 110CM |
| 270270008900000 | CATHETER ARTERIOGRAM |
| 270270009630000 | CATHETER CARDIAC DIAGNOSTIC |
| 270270009810000 | CATHETER CORONARY INFUSION |
| 270270045670000 | STENT CORONARY |
| 270270045750000 | STENT INTRACORONARY |
| 270270045830000 | STENT PTCA CORONARY FLEX |
| 270270045970000 | STENT PTCA COOK |
| 270270046990000 | SUCTION CORONARY |
| 270270101270000 | CATHETER CORONARY TEC 1.83MM |
| 270270101280000 | CATHETER CORONARY TEC 2.0MM |
| 270270110060000 | STENT DRUG ELUTING |
| 270270110170000 | STENT CORDIS CYPHER DRUG ELUTING |
| 270270110190000 | STENT TAXUS EXPRESS PACLITAXEL-ELUTING |
| 270270111670000 | STENT MEDTRONIC ENDEAVOR |
| 270270112010000 | STENT CORONARY WITH DELIVERY SYSTEM |
| 270272919850000 | CATHETER CORONARY ARTERY |
| 480480000030000 | ATHERECTOMY W/SUPPLIES |
| 480480000320000 | PTCA W/STENT EACH ADDL VESSEL |
| 480480000330000 | PTCA W/STENT SINGLE VESSEL |
| 480480000340000 | PTCA W/SUPPLIES |
| 480480002900000 | STENT PLACEMENT OF DRUG-ELUTING STENT PERC |
| 480480002910000 | STENT PLACEMENT OF DRUG-ELUTING STENT PERC ADDL |
| 480480929730000 | PERCUTANEOUS TRANSLUMINAL CORONARY THROMBECTOMY |
| 480480929750000 | THROMBOLYSIS CORONARY IC INFUSION |
| 480480929800000 | STENT PLACEMENT SINGLE VESSEL |
| 480480929810000 | STENT PLACEMENT EACH ADDL VESSEL |
| 480480929820000 | PTCA SINGLE VESSEL |
| 480480929840000 | PTCA EACH ADDL VESSEL |
| 480480929950000 | ATHERECTOMY SINGLE VESSEL |
| 480480929960000 | ATHERECTOMY EACH ADDL VESSEL |
| 480480934520000 | CATH LEFT HEART WITH LT VENTRICULOGRAPHY |
| 480480934530000 | CATH RIGHT & LEFT HEART WITH LT VENTRICULOGRAPHY |
| 480480934540000 | CATH PLACEMENT FOR CORONARY ANGIOGRAM |
| 480480934550000 | CATH PLACEMENT FOR CORONARY ANGIO BYPASS GRAFTS |
| 480480934560000 | CATH PLACEMENT FOR CORONARY ANGIO W/ RT HRT CATH |
| 480480934570000 | CATH PLACEMENT BYPASS GRAFT ANGIO W/ RT HRT CATH |
| 480480934580000 | CATH PLACE CORONARY ANGIO W/ LT HRT CATH/LT VENT |
| 480480934590000 | CATH PLACEMENT BYPASS GRAFT ANGIO W/ LT HRT CATH |
| 480480934600000 | CATH PLACEMENT CORONARY ANGIO W/ LT & RT HRT CATH |
| 480480934610000 | CATH PLACE BYPASS GRAFT ANGIO W/ LT & RT HRT CATH |
| 480480935640000 | INJ SELECT OPACIFICATION AORT/CORON/BYPASS GRFT |
| 480480935710000 | INTRAVASCULAR DOPPLER VELOCITY OR COLOR FLOW INIT |
| 480480935720000 | INTRAVASCULAR DOPPLER VELOCITY OR COLOR FLOW ADDL |
| 480481000180000 | CATH LEFT HEART W/LVA & CA |
| 480481000190000 | CATH LEFT HEART W/LVA CA & AO |
| 480481000200000 | CATH LEFT HEART W/LVA CA & BG |
| 480481000210000 | CATH LEFT HEART W/LVA CA BG & AO |
| 480481000350000 | R&LHC W/LVA & CA |
| 480481000360000 | R&LHC W/LVA CA & BG |
| 480481935080000 | CATH PLACE FOR CORONARY ANGIO W/O LEFT HEART CATH |
| 480481935400000 | INJ BYPASS GRAFTS |
| 480481935430000 | INJ LEFT ATRIAL/VENTRICULAR ANGIO |
| 480481935450000 | INJ CORONARY ANGIOGRAM |
| 980980929730000 | PF PERCUTANEOUS TRANSLUMINAL CORONARY THROMBECTOMY |
| 980980929750000 | PF THROMBOLYSIS CORONARY IC INFUSION |
| 980980929800000 | PF STENT PLACEMENT SINGLE VESSEL |
| 980980929810000 | PF STENT PLACEMENT EACH ADDL VESSEL |
| 980980929820000 | PF PTCA SINGLE VESSEL |
| 980980929840000 | PF PTCA EACH ADDL VESSEL |
| 980980929950000 | PF ATHERECTOMY SINGLE VESSEL |
| 980980934540000 | PF CATH PLACEMENT FOR CORONARY ANGIOGRAM |
| 980980934550000 | PF CATH PLACEMENT FOR CORONARY ANGIO BYPASS GRAFTS |
| 980980934580000 | PF CATH PLACE CORONARY ANGIO W/LT HRT CATH/LT VENT |
| 980980934600000 | PF CATH PLACE CORONARY ANGIO W/ LT & RT HRT CATH |
| 980980935080000 | PF CATH PLACE CORONARY ANGIO W/O LT HEART CATH |
| 980980935450000 | PF INJ CORONARY ANGIOGRAM |

# Table S1. Characteristics of Patients Hospitalized with Acute Myocardial Infarction by Hospital Risk-Standardized In-Hospital Cost Levels.

| **Variable** | **Total** | **Inpatient Cost Level** | | |  |
| --- | --- | --- | --- | --- | --- |
|  |  | **Low**  **($13,097-17,648)** | **Medium ($17,663-19,875)** | **High**  **($19,923-31,296)** | **p-value** |
| **Total No. of Patients** | 82,008 | 24,799 | 28,198 | 29,011 |  |
|  |  |  |  |  |  |
| **Demographics** |  |  |  |  |  |
| Age, median (IQR), years | 78  (71-85) | 78  (71-85) | 78  (71-85) | 78  (71-85) | **0.002** |
| Female (%) | 48.47 | 48.6 | 48.73 | 48.11 | 0.29 |
| Race/Ethnicity (%) |  |  |  |  | **<0.0001** |
| White | 75.6 | 73.0 | 79.3 | 74.2 |  |
| Black | 7.9 | 7.4 | 7.2 | 9.1 |  |
| Hispanic | 0.9 | 0.78 | 0.9 | 0.9 |  |
| Other | 15.6 | 18.9 | 12.6 | 15.8 |  |
| **Comorbidities (%)** |  |  |  |  |  |
| Disorders of lipid metabolism | 64.1 | 66.6 | 64.7 | 61.4 | **<0.0001** |
| Coronary atherosclerosis and other heart disease | 83.9 | 83.7 | 84.2 | 83.8 | 0.20 |
| History of Acute myocardial infarction | 1.3 | 1.4 | 1.3 | 1.3 | 0.40 |
| Peripheral and visceral atherosclerosis | 14.5 | 15.7 | 14.3 | 13.8 | **<0.0001** |
| Aortic, peripheral, and visceral artery aneurysms | 3.1 | 3.2 | 3.1 | 3.1 | 0.73 |
| Aortic and peripheral arterial embolism or thrombosis | 0.4 | 0.5 | 0.4 | 0.5 | 0.55 |
| Transient cerebral ischemia | 0.6 | 0.5 | 0.6 | 0.6 | 0.42 |
| Cardiac dysrhythmias | 41.1 | 42.7 | 40.5 | 40.2 | **<0.0001** |
| Cardiac arrest and ventricular fibrillation | 5.3 | 5.7 | 5.2 | 5.2 | **0.02** |
| History of VT or VF | 43.3 | 45 | 42.7 | 42.3 | **<0.0001** |
| History of peripheral vascular disease | 16.9 | 18 | 16.6 | 16.3 | **<0.0001** |
| History of CABG | 13.7 | 13.1 | 14.0 | 13.9 | **0.0039** |
| History of PCI | 23.0 | 21.7 | 23.5 | 23.6 | **<0.0001** |
| Congestive heart failure | 0.7 | 0.5 | 0.7 | 0.8 | **0.0008** |
| Valvular disease | 0.3 | 0.2 | 0.3 | 0.3 | **0.03** |
| Pulmonary circulation disease | 0.1 | 0.1 | 0.2 | 0.1 | 0.93 |
| Peripheral vascular disease | 17.4 | 18.5 | 17.1 | 16.7 | **<0.0001** |
| Paralysis | 2.2 | 2.1 | 2.5 | 2.1 | **0.0018** |
| Other neurological disorders | 9.1 | 9.3 | 9.6 | 8.5 | **<0.0001** |
| Chronic pulmonary disease | 25.5 | 25.3 | 25.4 | 25.6 | 0.65 |
| Diabetes | 32.3 | 31.8 | 33 | 32 | **0.0037** |
| Diabetes w/chronic complications | 7 | 7.6 | 7 | 6.6 | **<0.0001** |
| Hypothyroidism | 16.3 | 17.1 | 16.4 | 15.7 | **<0.0001** |
| Renal failure | 29.7 | 30.8 | 29.7 | 28.9 | **<0.0001** |
| Liver disease | 1.2 | 1.2 | 1.2 | 1.1 | 0.64 |
| Lymphoma | 0.8 | 0.8 | 0.8 | 0.7 | 0.36 |
| Metastatic cancer | 1.3 | 1.3 | 1.3 | 1.4 | 0.37 |
| Solid tumor w/out metastasis | 2.1 | 2.1 | 2.1 | 2.1 | 0.94 |
| Rheumatoid arthritis/collagen vascular disease | 3.2 | 3.7 | 3.2 | 2.8 | **<0.0001** |
| Coagulopathy | 7.4 | 7.3 | 7.1 | 7.8 | **0.0029** |
| Obesity | 11.3 | 12.3 | 11.3 | 10.4 | **<0.0001** |
| Weight loss | 4.4 | 4.2 | 5 | 4.1 | **<0.0001** |
| Fluid and electrolyte disorders | 29 | 30.6 | 27.9 | 28.6 | **<0.0001** |
| Chronic blood loss anemia | 1.4 | 1.5 | 1.3 | 1.5 | **0.0197** |
| Deficiency anemias | 25.6 | 25.7 | 25.6 | 25.4 | 0.70 |
| Alcohol abuse | 1.8 | 1.9 | 1.8 | 1.6 | 0.10 |
| Drug abuse | 0.5 | 0.7 | 0.5 | 0.4 | **<0.0001** |
| Psychoses | 2.4 | 2.3 | 2.1 | 2.7 | **0.0002** |
| Depression | 8.5 | 9.5 | 8.8 | 7.2 | **<0.0001** |
| Hypertension | 77.9 | 78.6 | 77.7 | 77.6 | **0.0071** |
| CABG: coronary artery bypass graft; IQR: interquartile range; PCI: percutaneous coronary intervention; VF: ventricular fibrillation; VT: ventricular tachycardia | | | | | |

# Table S2. In-Hospital Resource Utilization: Mean Per-Patient Cost in Each Department.

| **Department** |  | **Inpatient Cost Level ($)** |  | **p-value^1^** | **Significant Pair-wise Comparison^2^** |
| --- | --- | --- | --- | --- | --- |
|  | **Low (N=24,799)** | **Medium (N=28,198)** | **High (N=29,011)** |  |  |
| Cardiac Procedures | 2008 | 2153 | 2074 | <.0001 | High vs. Med. and Low vs. Med. |
| Other Cardiology | 211 | 230 | 655 | <.0001 | All pairs |
| ICU/CCU | 2339 | 2876 | 4285 | <.0001 | All pairs |
| Other Room And Board | 3716 | 4047 | 3856 | <.0001 | All pairs |
| Lab | 974 | 1119 | 1235 | <.0001 | All pairs |
| Operating Room | 977 | 1212 | 1277 | <.0001 | All pairs |
| Pharmacy | 1408 | 1572 | 2578 | <.0001 | All pairs |
| Radiology | 610 | 673 | 771 | <.0001 | All pairs |
| Respiratory | 400 | 476 | 592 | <.0001 | All pairs |
| Supply | 2435 | 3031 | 3509 | <.0001 | All pairs |
| Other | 994 | 1071 | 1542 | <.0001 | All pairs |

ICU: intensive care unit; CCU: coronary care unit

^1^p-value for Wilcoxon analysis

^2^Using Dwass, Steel, Critchlow-Fligner (DSCF) method for multiple comparison analysis.

# Table S3. In-Hospital Resource Utilization: Mean per Patient Number of Items

# Ordered in Each Department.

| **Department** | **Cost Level** | | |
| --- | --- | --- | --- |
|  | **Low (N=24,799)** | **Medium (N=28,198)** | **High (N=29,011)** |
| Cardiac Procedures | 1.6 | 1.8 | 1.7 |
| Other Cardiology | 3.4 | 3.6 | 4.0 |
| ICU/CCU | 1.3 | 1.6 | 2.3 |
| Other Room And Board | 3.8 | 4.0 | 3.8 |
| Lab | 41.4 | 45.6 | 51.0 |
| Operating Room | 1.4 | 1.8 | 1.9 |
| Pharmacy | 79.5 | 86.1 | 98.5 |
| Radiology | 4.5 | 5.0 | 5.6 |
| Respiratory | 6.9 | 7.9 | 8.6 |
| Supply | 14.9 | 18.7 | 24.7 |
| Other | 8.6 | 10.2 | 12.0 |

ICU: intensive care unit; CCU: coronary care unit

# Table S4. Patient Costs by In-Hospital Cost Ranks and Vital Status.

| **In-hospital Cost Rank** | **Death** | **Number of Patients** | **Mean** | **Standard Deviation** | **Median** | **Q1** | **Q3** |
| --- | --- | --- | --- | --- | --- | --- | --- |
| 1 | Survivor | 22755 | 16001.2 | 13562.49 | 12357.93 | 8032.98 | 17943.78 |
| 1 | Dead | 2044 | 15222.99 | 17449.41 | 9719.33 | 4709.77 | 18333 |
| 2 | Survivor | 25865 | 18311.38 | 15425.12 | 13850.35 | 9247.68 | 20882.37 |
| 2 | Dead | 2333 | 17899.29 | 20229.28 | 11189.61 | 5328.57 | 21748.22 |
| 3 | Survivor | 26609 | 21841.55 | 18872.66 | 15880.77 | 10631.33 | 25455.08 |
| 3 | Dead | 2402 | 22097.77 | 24276.24 | 13628.91 | 6306.26 | 26888.89 |

# Table S5. Mean Per Patient Post-Acute-Care Payment at Different Care Settings.

|  | **Inpatient Cost Level ($)** | | | **p-value^1^** | **Significant**  **Pair-wise**  **Comparison^2^** |
| --- | --- | --- | --- | --- | --- |
| **Care Setting** | **Low (N=22318)** | **Medium (N=25380)** | **High**  **(N=21029)** |  |  |
| Emergency Department | 81 | 80 | 74 | 0.0734 | NA |
| Home Health Agency | 255 | 249 | 229 | <.0001 | High vs. Low  High vs. Med |
| Hospice | 117 | 94 | 111 | 0.0002 | Med vs. Low |
| Miscellaneous | 182 | 201 | 201 | 0.0456 | Med vs. Low |
| Non-Acute Inpatient Settings | 533 | 649 | 714 | <.0001 | High vs. Low  Med vs. Low |
| Observation Stay | 55 | 59 | 64 | 0.0077 | High vs. Low |
| Other Outpatient Settings | 319 | 314 | 286 | <.0001 | High vs. Low  Med vs. Low |
| Outpatient Physician Visits | 139 | 138 | 137 | <.0001 | All pairs |
| Readmission | 1534 | 1633 | 1596 | 0.0013 | High vs. Low  Med vs. Low |
| Skilled Nursing Facility | 1535 | 1542 | 1303 | <.0001 | High vs. Low  High vs. Med |

*Includes payments for readmission facilities and physicians

^1^p-value for Wilcoxon analysis

^2^Using Dwass, Steel, Critchlow-Fligner (DSCF) method for multiple comparison analysis.

# Table S6. Percentage of Patients Using Various Post-Acute-Care Services

# by Cost Groups.

|  | **Cost Level (%)** | | |
| --- | --- | --- | --- |
| **Care Setting** | **Low (N=22318)** | **Medium (N=25380)** | **High**  **(N=21029)** |
| Emergency Department | 20.4 | 20.8 | 20.3 |
| Home Health Agency | 20.5 | 20.0 | 18.5 |
| Hospice | 3.9 | 3.2 | 3.6 |
| Miscellaneous | 35.6 | 36.4 | 36.0 |
| Non-Acute Inpatient Settings | 3.5 | 4.2 | 4.5 |
| Observation Stay | 3.6 | 3.5 | 3.2 |
| Other Outpatient Settings | 56.2 | 53.1 | 52.5 |
| Outpatient Physician Visits | 65.7 | 63.4 | 62.8 |
| Readmission* | 34.4 | 36.3 | 36.7 |
| Skilled Nursing Facility | 19.5 | 19.4 | 17.3 |

*Includes payments for readmission facilities and physicians.

# Figure S1. Flowchart of Exclusion Criteria


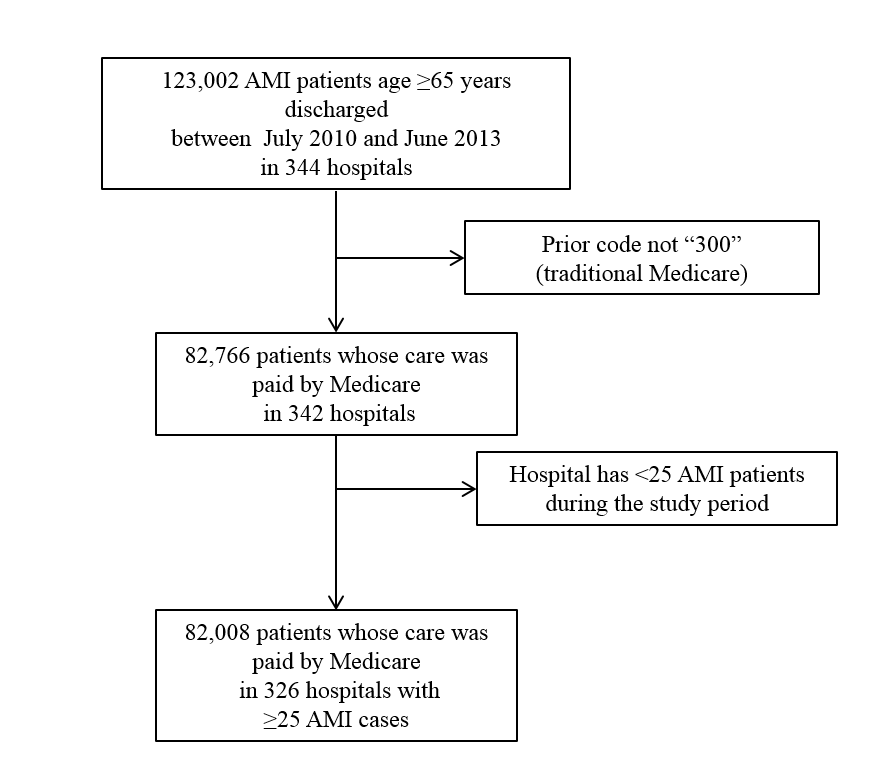


# Figure S2. Number of Items Ordered during a Hospitalization by In-Hospital Cost Tertile.

# Figure S3. Mean In-hospital Resource Utilization per User by In-Hospital Cost Tertiles.

# Figure S4. Relative Contribution of Service Categories to In-Hospital Costs by In-Hospital Cost Tertiles.

# Figure S5. Correlation Between Hospital Risk-Standardized In-Hospital Cost ($) and Hospital Risk-Standardized Post-Acute Payments ($).


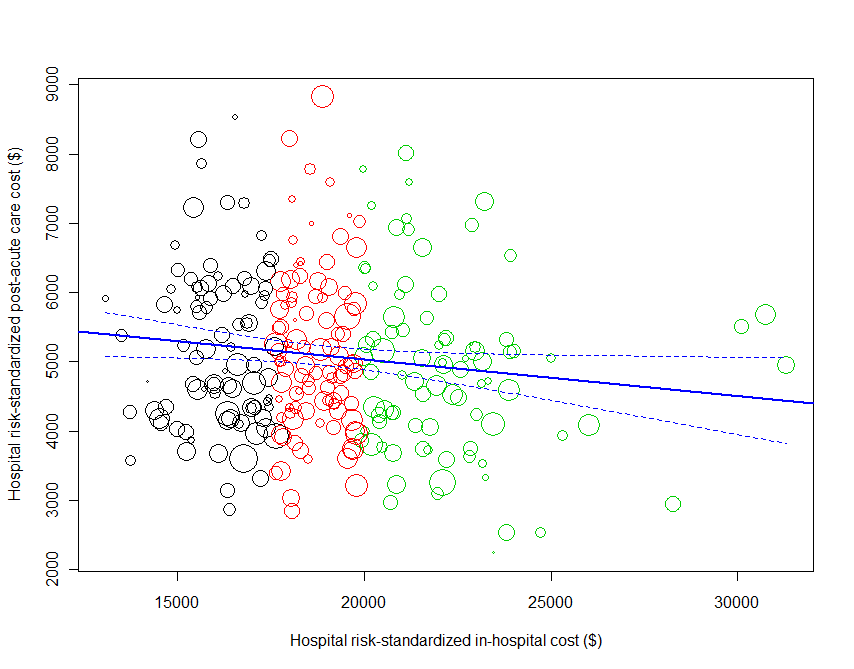


Each circle represents one hospital in our analysis. The colors of the circles indicate the in-hospital risk-standardized cost groups: black (low), red (medium), green (high). The size of the circles is proportional to the logarithm of the AMI patient volume/25. The line of best fit (trend line; dashed lines represent 95% confidence interval) is shown indicating the association between the hospital risk-standardized in-hospital cost ($) and hospital risk-standardized post-acute care payments ($).

# Figure S6a. In-Hospital Spending Among Survivors.

# Figure S6b. In-Hospital Spending Among the Expired.

# Figure S7. Percentage of Patients Using Different Post-Acute-Care Services by In-Hospital Cost Tertiles.

# Figure S8. Mean User Post-Acute Payments by In-hospital Cost Tertiles.

# Figure S9. Relative Contribution of Service Categories to Post-Acute Payments by In-Hospital Cost Tertiles.
